# Supplementary material for: Hakai is required for stabilization of core components of the m6A mRNA methylation machinery
Source: Nat Commun. 2021 Jun 18;12:3778. doi: 10.1038/s41467-021-23892-5 (PMC8213727; doi:10.1038/s41467-021-23892-5)
Supplement: Supplementary file 1 — Supplementary Information [file 41467_2021_23892_MOESM1_ESM.pdf]

Supplementary information

Hakai is required for stabilization of core components of the m^{6}A mRNA methylation machinery

Praveen Bawankar[#], Tina Lence[#], Chiara Paolantoni[#], Irmgard U. Haussmann, Migle Kazlauskiene, Dominik Jacob, Jan B. Heidelberger, Florian Richter, Mohanakarthik P. Nallasivan, Violeta Morin, Nastasja Kreim, Petra Beli, Mark Helm, Martin Jinek, Matthias Soller[*] and Jean-Yves Roignant[*]

[#] These authors contributed equally

[*] Correspondence to jean-yves.roignant@unil.ch, M.Soller@bham.ac.uk

Supplementary Figures

Supplementary Figure 1

Supplementary Figure 2

Supplementary Figure 3

Supplementary Figure 4

Supplementary Figure 5

Supplementary Figure 6

Supplementary Figure 7

Supplementary Figure 8

Supplementary Figure 9

Supplementary References

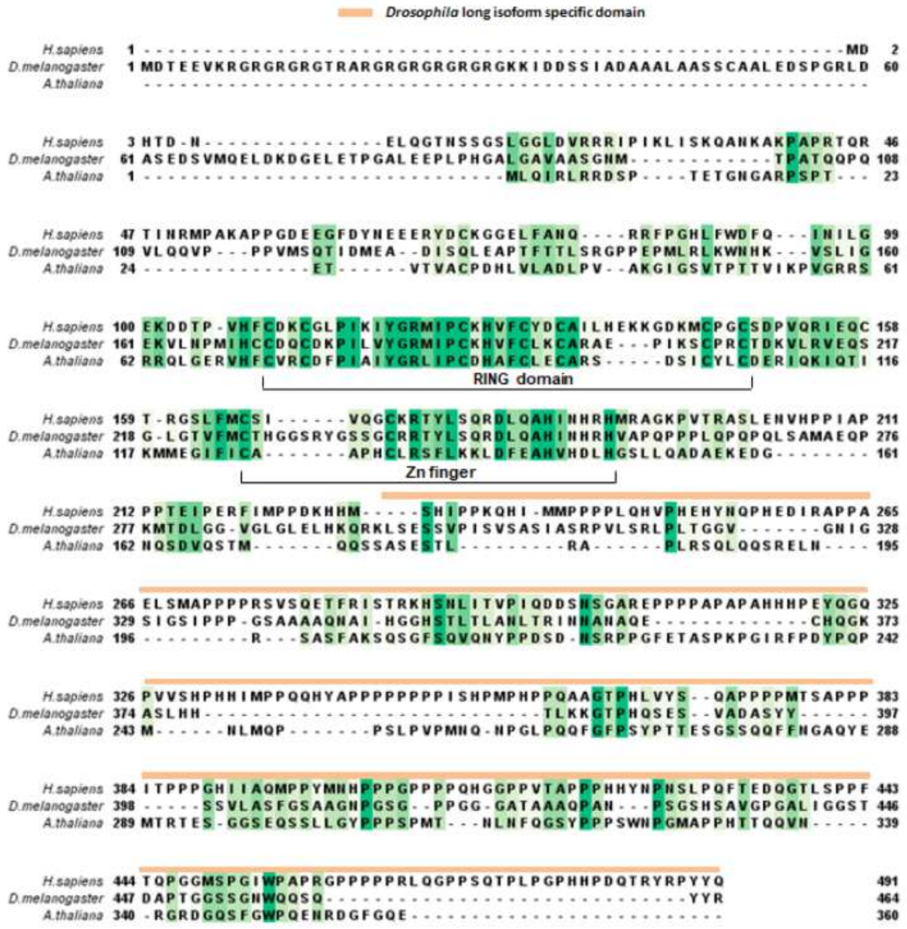

Supplementary Figure 1. Hakai protein alignment.

Alignment of Hakai protein sequences from *Drosophila melanogaster* (Hakai_Dm: M9PBE2-1), *Arabidopsis thaliana* (Hakai_At:Q9LFC0) and *Homo sapiens* (HAKAI_Hs: Q75N03-1). (6,4% identity, 64 aa similarities). Protein alignments were generated in Jalview 2.10.5 (Waterhouse et al. 2009) with ClustalO version1.2.4 using default settings (Larkin et al. 2007). Colored by degree of amino acid conservation in each column with a 30 % cut off threshold. The orange line indicates the C-terminal region specific of the long isoforms.

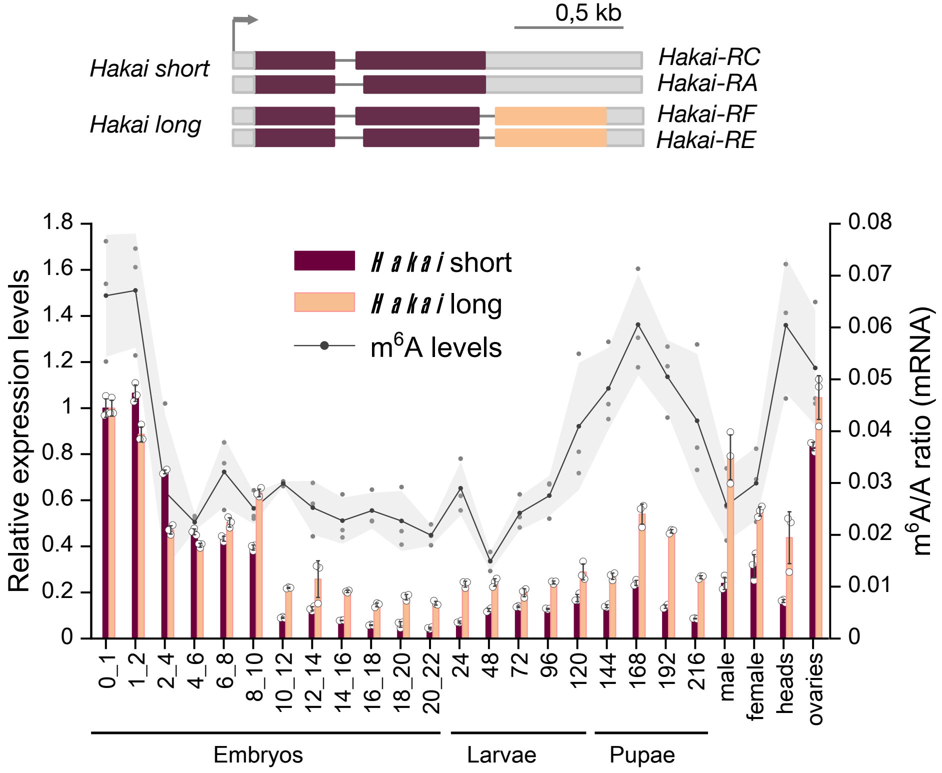

Supplementary Figure 2. Developmental expression of *Hakai* transcript isoforms.

(Top) Schematic representation of two short and two long *Hakai* transcript isoforms generated by alternative splicing. 5'UTR and 3'UTR sequences are shown in grey and CDS in colors, connecting lines represent introns. (Bottom) Relative expression of *Hakai* short and long transcript isoforms during *D. melanogaster* developmental stages and in adult female heads and in ovaries, analyzed by qRT-PCR. Levels of m^6A modification were analyzed using LC-MS/MS (grey line). All *Hakai* isoforms and m^6A levels are enriched during first hours of embryogenesis, during early pupation as well as in adult heads and ovaries. Bars represent the mean with standard deviation (SD) of three technical measurements from one biological replicate. Line junctions display mean with standard deviation (SD) of three technical measurements from three biological replicates. Source data for m^6A measurement and qPCR are provided as a Source Data file.

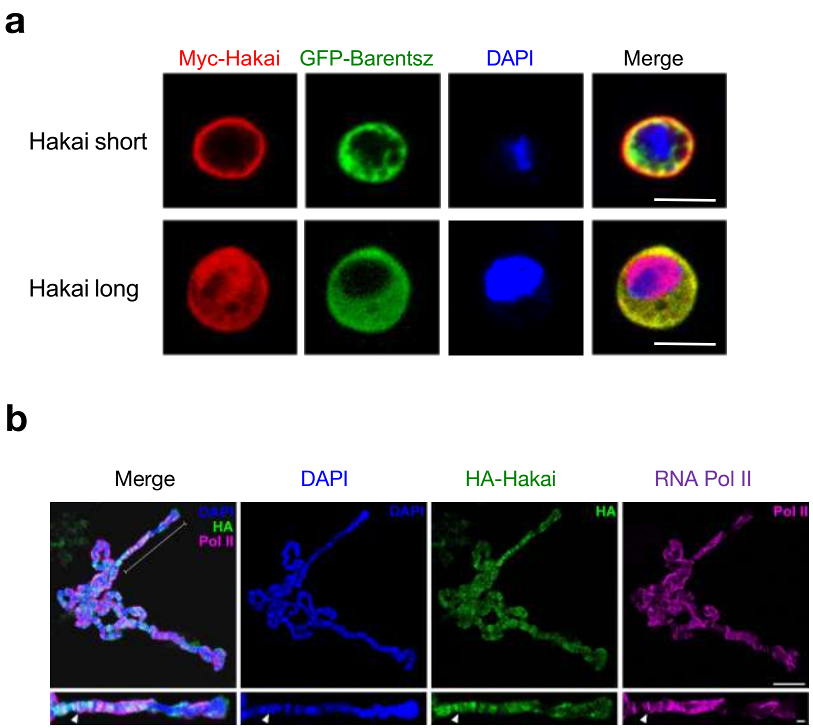

Supplementary Figure 3. The long Hakai isoform localizes to the nucleus

(**a**) Immunostaining of Myc-tagged Hakai short and long protein isoforms overexpressed in BG3 cells. GFP-tagged Barentsz protein served as a cytoplasmic marker. DAPI staining is shown in blue. The short Hakai isoform localizes strictly to the cytoplasm, whereas the long isoform localizes to both cellular compartments with the enrichment in the nucleus. Scale bars, 10 µm

(**b**) Immunostaining of Polytene chromosomes of Salivary glands. HA-Hakai (green) colocalizes partially with RNA Pol II (magenta). The images shown are representative of three biological replicates. Scale bars: (top) 20 µm, (bottom) 1 µm.

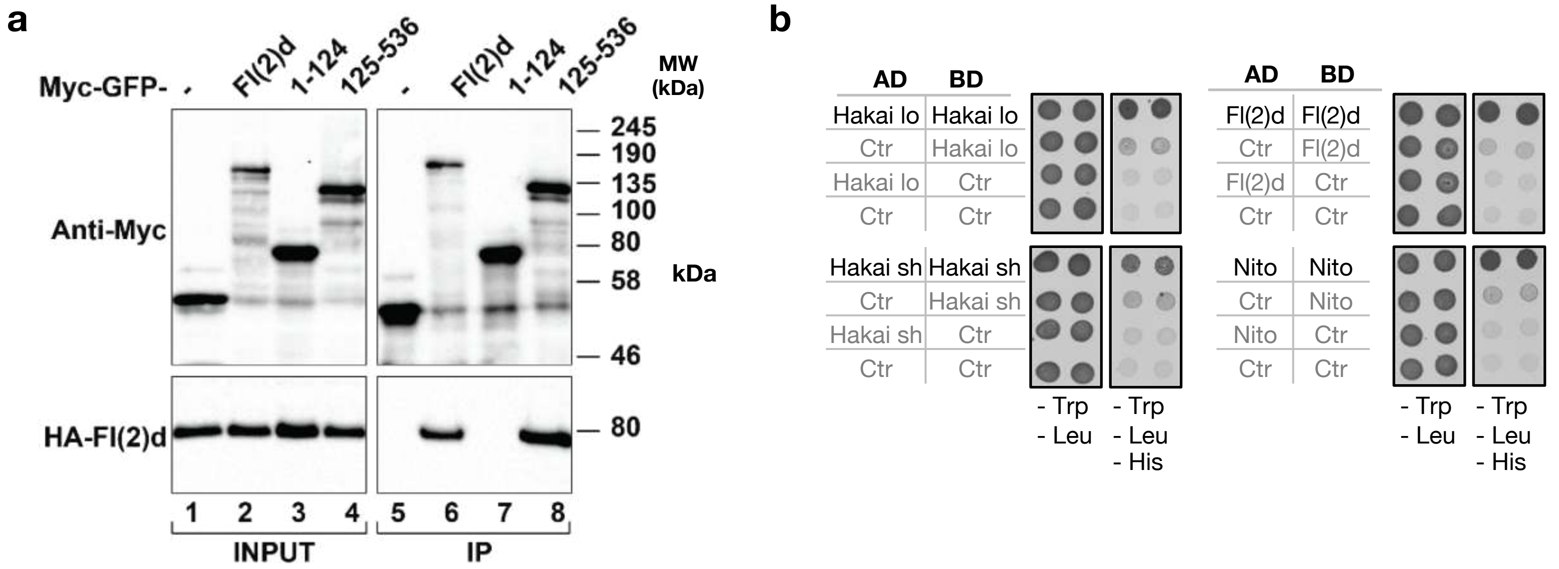

Supplementary Figure 4. Hakai, Nito and Fl(2)d self-interact.

(**a**) Co-immunoprecipitation experiments were carried out with lysates prepared from S2R+ cells transfected with Myc-GFP-tagged Fl(2)d (full length or fragments) (A) and HA-tagged Fl(2)d. In control lanes, S2R+ cells were transfected with Myc-GFP alone and an identical HA-containing protein. Extracts were incubated with magnetic agarose GFP binder beads and immunoblotted using anti-Myc and anti-HA antibodies, as indicated. Two percent of input was loaded. The experiment was performed in the presence of RNase A.

(**b**) Yeast-two-hybrid assay to investigate Hakai, Nito and Fl(2)d homo-dimerization. Proteins were cloned in yeast expression vectors and fused with either Gal4-DNA binding domain (BD) or Gal4-DNA activation domain (AD). Indicated combinations of vectors were co-expressed in yeast and empty vectors encoding only activation or binding domain were used as control (Ctr). Recovered colonies were spotted on plates lacking Leucin and Tryptophan (-Leu, -Trp) as well as selection plates lacking amino acids Leucin, Tryptophan and Histidine (-Leu, -Trp, -His). Hakai, Fl(2)d and Nito all homo dimerize.

Source Data for Western blots and the yeast two hybrid assay are provided as a Source Data file.

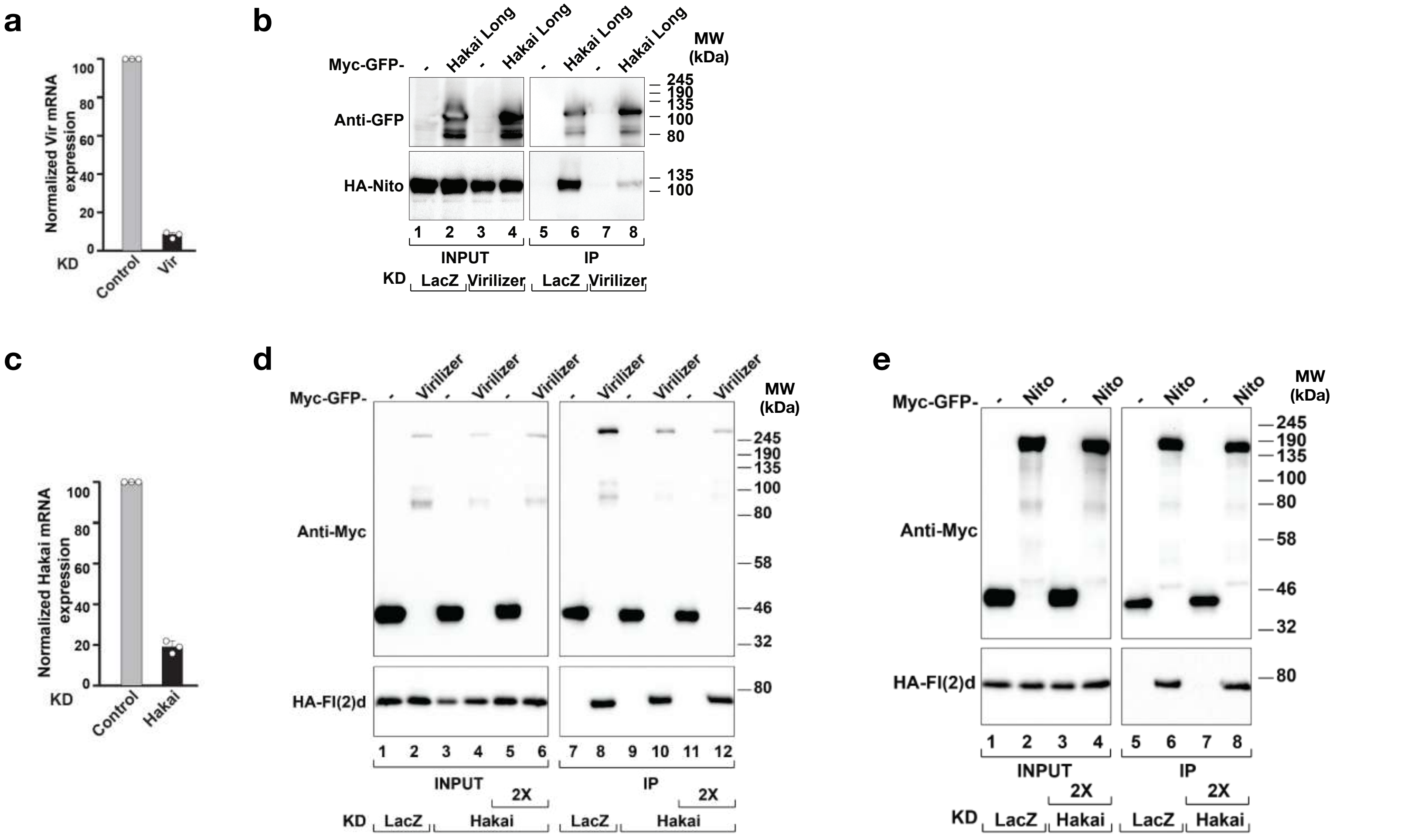

Supplementary Figure 5. Dependency on Hakai and Virilizer for molecular interactions within MACOM.

(**a**) Relative expression levels of *Vir* shown as a validation of its KD efficiency. The mean with standard error (SE) of three technical measurements is shown.

(**b**) Co-immunoprecipitation experiments were carried out with lysates prepared from S2R+ cells transfected with Myc-GFP-tagged Hakai-long and HA-tagged Nito upon control (*LacZ*) or *vir* KDs. In control lanes, S2R+ cells were transfected with Myc-GFP alone and an identical HA-containing protein. Extracts were incubated with magnetic agarose GFP binder beads and immunoblotted using anti-GFP and anti-HA antibodies. Two percent of input was loaded.

(**c**) Relative expression levels of *Hakai* shown as a validation of its KD efficiency. The mean with standard error (SE) of three technical measurements is shown.

(**d, e**) Co-immunoprecipitation experiments were carried out with lysates prepared from S2R+ cells transfected with Myc-GFP-tagged Vir (d) or Myc-GFP-tagged Nito (e) and HA-tagged Fl(2)d upon control (*LacZ*) or *Hakai* KDs. In control lanes, S2R+ cells were transfected with Myc-GFP alone and an identical HA-containing protein. Extracts were incubated with magnetic agarose GFP binder beads and immunoblotted using anti-Myc and anti-HA antibodies. Two percent of input was loaded. Note that twice the amount of plasmids was transfected to compensate for the decreased protein levels. Images shown are representative of two biological replicates.

Source Data for Western blots are provided as a Source Data file.

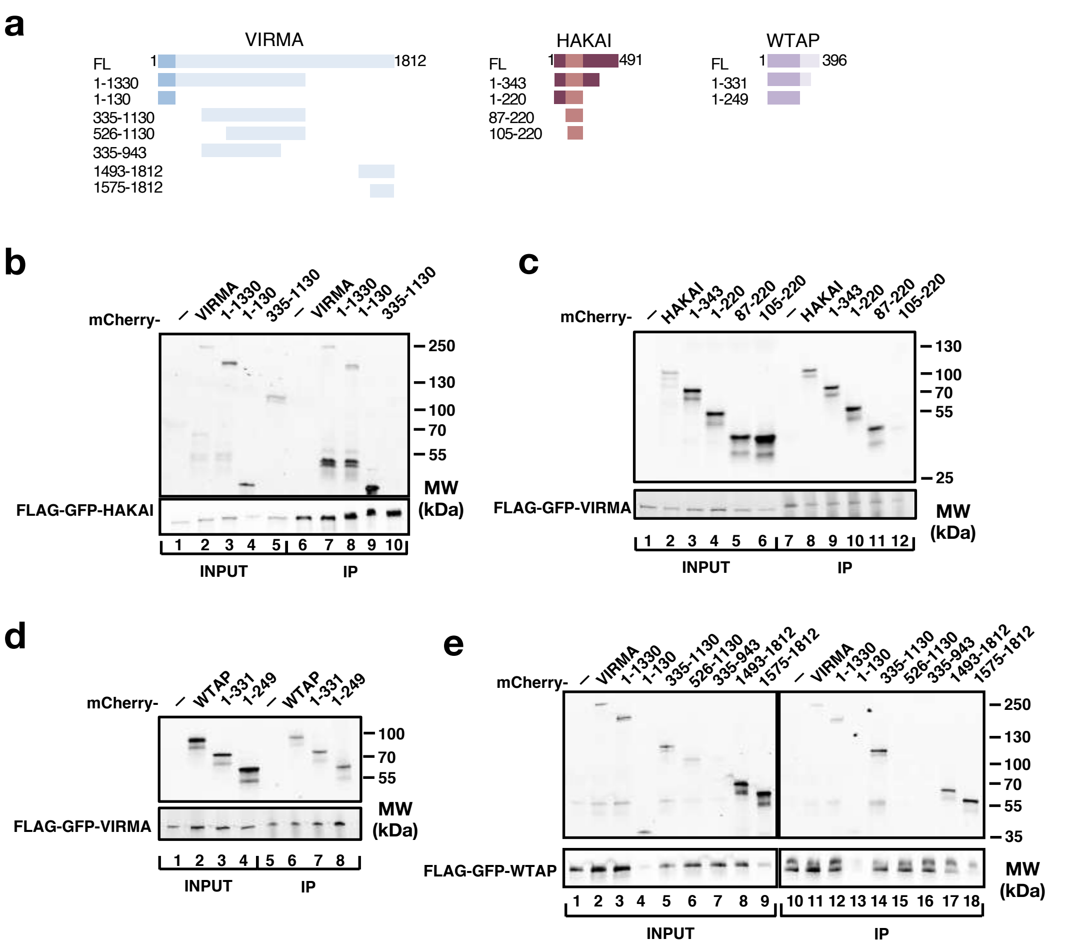

Supplementary Figure 6. VIRMA serves as an interaction platform for the assembly of HAKAI and WTAP into the MACOM complex in humans.

(**a**) Schematic representation of proteins and protein fragments used for human co-immunoprecipitation assays.

(**b-e**) Co-IP assays were carried out with lysates prepared from HEK293T cells transfected with (**b**) His6-FLAG3-eGFP-HAKAI (full-length) and His6-HA3-mCherry-VIRMA (full-length or indicated truncations); (**c**) His6-FLAG3-eGFP-VIRMA (full-length) and His6-HA3-mCherry-HAKAI (full-length or indicated truncations); (**d**) His6-FLAG3-eGFP-VIRMA (full-length) and His6-HA3-mCherry-WTAP (full-length or indicated truncations). Extracts were immunoprecipitated with magnetic anti-FLAG M2 beads and analysed based on fluorescence. 2% of input was loaded. (**e**) His6-FLAG3-eGFP-WTAP (full-length) and His6-HA3-mCherry-VIRMA (full-length or indicated truncations). The experiment was performed in the presence of RNase A.

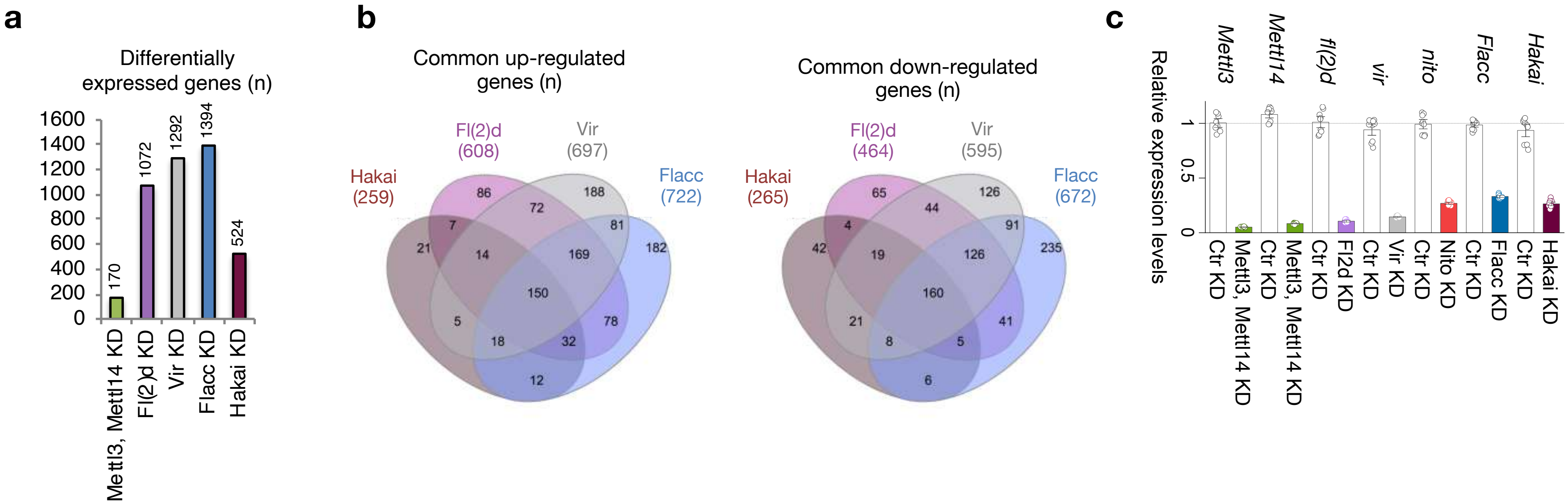

Supplementary Figure 7. Hakai regulates m^{6}A-dependent gene expression.
(**a**) Number of differentially expressed genes upon knockdown of indicated proteins (FDR<0.05). N = three biological replicates.
(**b**) Common differentially up-regulated (left) or down-regulated (right) genes upon knockdown of indicated proteins (FDR<0.05).
(**c**) Relative expression levels of indicated transcripts displaying validation of KD efficiency. The mean with standard error (SE) of three biological replicates and three technical measurements is shown.
Source Data for RNA-seq and qPCR are provided as a Source Data file.

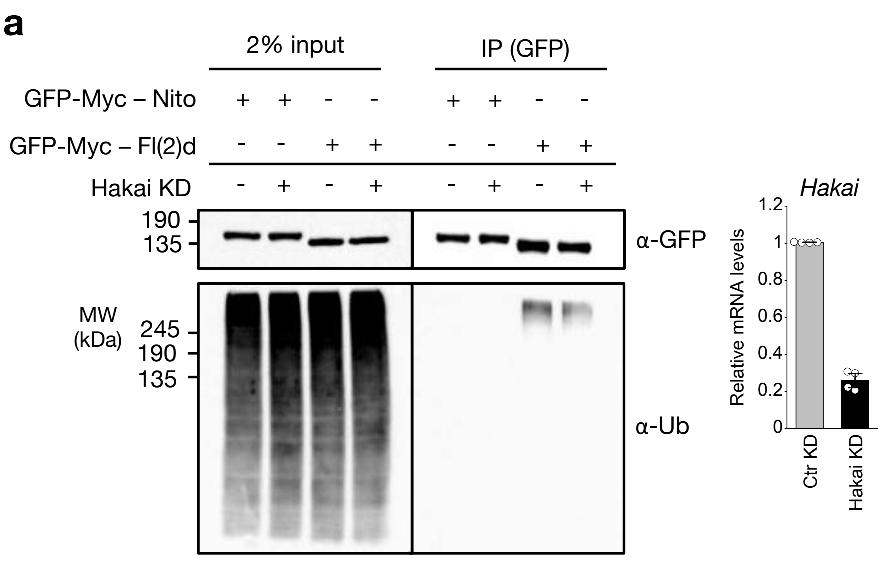

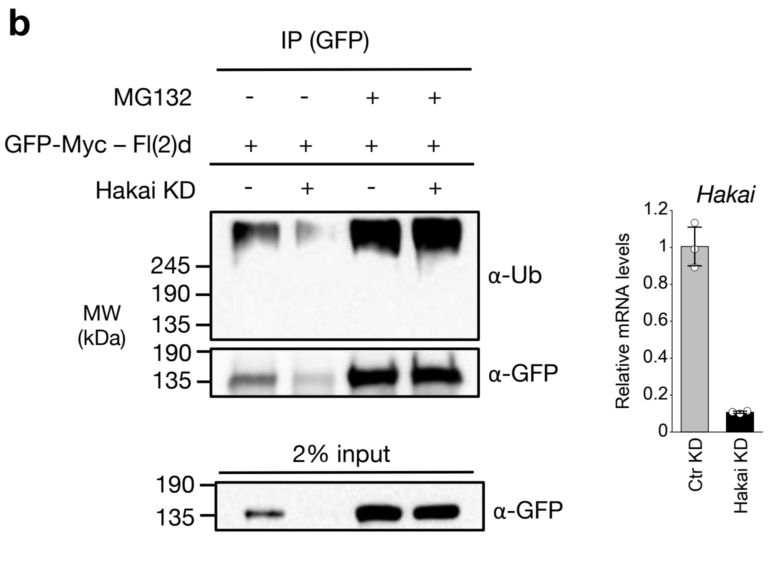

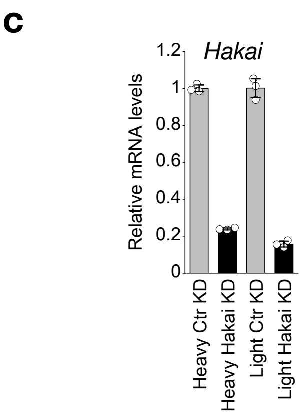

Experiment	Heavy labelled cells	Light labelled cells
Replicate 1 (Fwd)	**Hakai KD**	Control
Replicate 2 (Rev)	Control	**Hakai KD**

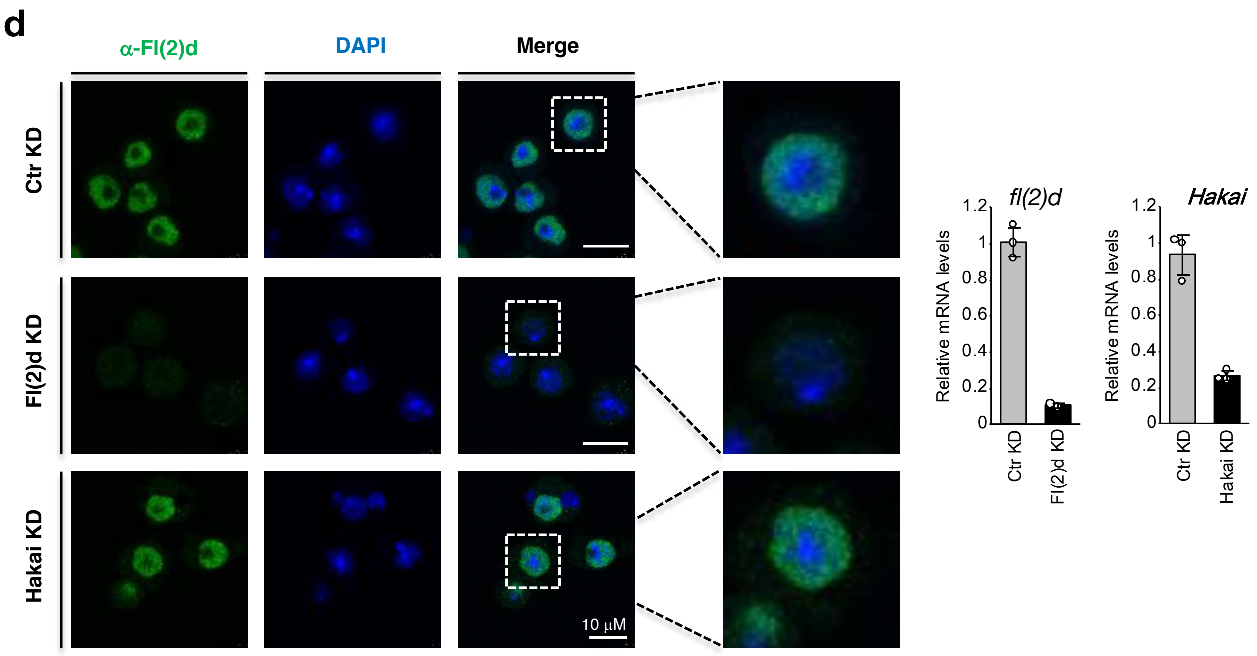

Supplementary Figure 8. Hakai does not ubiquitinate MACOM components in S2R+ cells and is not required for Fl(2)d subcellular localization.
(**a**) Ubiquitination analysis of GFP-tagged Nito and Fl(2)d proteins upon control condition and Hakai depletion. GFP-tagged proteins were immunoprecipitated using anti-GFP-coupled beads under stringent urea conditions and analyzed by western blot. (left). Relative expression levels of *Hakai* shown as a validation of its KD efficiency. The mean with standard error (SE) of four technical measurements is shown (right). Fl(2)d, but not Nito, is ubiquitinated.
(**b**) Ubiquitination analysis of GFP-tagged Fl(2)d protein upon control condition and Hakai depletion, with or without proteasome inhibitor MG132. GFP-tagged Fl(2)d was immunoprecipitated using anti-GFP-coupled beads under stringent urea conditions and analyzed by western blot (left). Relative expression levels of *Hakai* shown as a validation of its KD efficiency. The mean with standard error (SE) of three technical measurements is shown (right). Fl(2)d stability and ubiquitination are decreased upon Hakai depletion and rescued by inhibition of proteasomal degradation.
(**c**) Relative expression levels of *Hakai* are shown as a validation of its KD efficiencies for the ubiquitylome and proteome in S2R+ cells. The mean with standard error (SE) of three technical measurements is shown (top). Experimental set-up of double labelling SILAC experiment for replicate 1 (Fwd) and replicate 2 (Rev) (below).
(**d**) Immunostaining of endogenous Fl(2)d protein (green) in S2R+ cells upon depletion of *Fl(2)d* or *Hakai* and in control condition. DAPI staining is shown in blue. Fl(2)d localization is unperturbed upon Hakai depletion in S2R+ cells. Scale bars, 10 µm. Right, relative expression levels of *Fl(2)d* and *Hakai* are shown as a validation of their KD efficiencies. The mean with standard error (SE) of three technical measurements is shown.
Source Data for Western blots and qPCR are provided as a Source Data file.

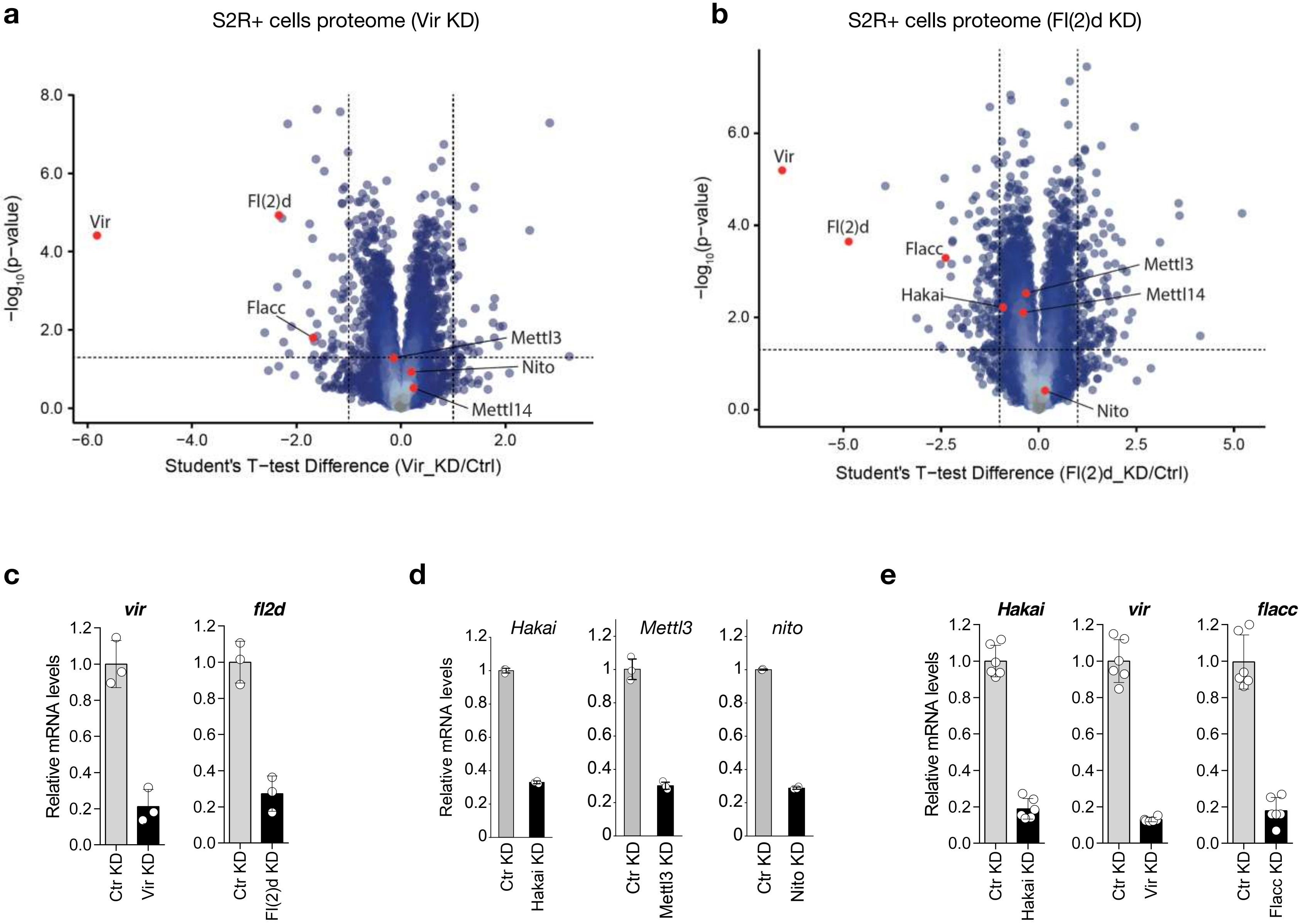

a
S2R+ cells proteome (Vir KD)
−log10(p−value)
Student's T−test Difference (Vir_KD/Ctrl)
Vir
Fl(2)d
Flacc
Mettl3
Nito
Mettl14
b
S2R+ cells proteome (Fl(2)d KD)
−log10(p−value)
Student's T−test Difference (Fl(2)d_KD/Ctrl)
Vir
Fl(2)d
Flacc
Hakai
Mettl3
Mettl14
Nito
c
vir
fl2d
Relative mRNA levels
Ctr KD
Vir KD
Fl(2)d KD
d
Hakai
Mettl3
nito
Relative mRNA levels
Ctr KD
Hakai KD
Mettl3 KD
Nito KD
e
Hakai
vir
flacc
Relative mRNA levels
Ctr KD
Hakai KD
Vir KD
Flacc KD

Supplementary Figure 9. Vir and Fl(2)d control the stability of other MACOM components.
(**a-b**) Quantitative mass spectrometry analysis of Vir-dependent proteome (A) or Fl(2)d-dependent proteome (B) in S2R+ cells. Label free quantification on four replicate experiments was performed to identify significantly upregulated and down regulated proteins. MACOM component proteins are highlighted in red. The threshold was set to a FDR of 0.05 (black dashed line y-axis) and to a T-test difference of ±1 (black dashed line x-axis).
(**c**) Relative expression levels of *fl(2)d* and *vir* are shown as a validation of their KD efficiencies for the proteome in S2R+ cells. The mean with standard deviation of three technical measurements is shown.
(**d**) Relative expression levels of *Hakai*, *Mettl3* and *nito* are shown as a validation of their KD efficiencies for the analysis of Fl(2)d protein levels. The mean with standard error (SE) of three technical measurements is shown.
(**e**) Relative expression levels of *Hakai*, *vir* and *Flacc* are shown as a validation of their KD efficiencies for the analysis of Fl(2)d protein levels. The mean with standard deviation of two biological replicates and three technical measurements is shown.
Source Data for qPCR are provided as a Source Data file.

Supplementary References

1. Larkin MA, Blackshields G, Brown NP, Chenna R, McGettigan PA, McWilliam H, Valentin F, Wallace IM, Wilm A, Lopez R et al. 2007. Clustal W and Clustal X version 2.0. *Bioinformatics* **23**: 2947-2948.
2. Waterhouse AM, Procter JB, Martin DM, Clamp M, Barton GJ. 2009. Jalview Version 2--a multiple sequence alignment editor and analysis workbench. *Bioinformatics* **25**: 1189-1191.
