# Supplementary material for: Hakai is required for stabilization of core components of the m6A mRNA methylation machinery
Source: Nat Commun. 2021 Jun 18;12:3778. doi: 10.1038/s41467-021-23892-5 (PMC8213727; doi:10.1038/s41467-021-23892-5)
Supplement: Supplementary file 3 — Description of Additional Supplementary Files [file 41467_2021_23892_MOESM3_ESM.docx]

File Name: Supplementary Data 1
Description: Mass spectrometry analysis of proteins pulled down after immunoprecipitation of Hakai long isoform in S2R+ cells

File Name: Supplementary Data 2
Description: Mass spectrometry analysis of ubiquitylation sites after pull down of GFP-Fl(2)d or GFP-Nito in S2R+ cells

File Name: Supplementary Data 3
Description: Mass Spectrometry analysis of Hakai-dependent ubiquitinated proteins in S2R+ cells

File Name: Supplementary Data 4
Description: Proteome analysis upon Hakai KD in S2R+ cells

File Name: Supplementary Data 5
Description: List of oligonucleotide used in this study
