# Supplementary material for: Hakai is required for stabilization of core components of the m6A mRNA methylation machinery
Source: Nat Commun. 2021 Jun 18;12:3778. doi: 10.1038/s41467-021-23892-5 (PMC8213727; doi:10.1038/s41467-021-23892-5)
Supplement: Supplementary file 9 — Reporting Summary [file 41467_2021_23892_MOESM9_ESM.pdf]

nature research

Corresponding author(s): Matthias Soller, Jean-Yves Roignant

Last updated by author(s): 2021/05/03

Reporting Summary

Nature Research wishes to improve the reproducibility of the work that we publish. This form provides structure for consistency and transparency in reporting. For further information on Nature Research policies, see our Editorial Policies and the Editorial Policy Checklist.

Statistics

For all statistical analyses, confirm that the following items are present in the figure legend, table legend, main text, or Methods section.

n/a	Confirmed	
☐	☒	The exact sample size (*n*) for each experimental group/condition, given as a discrete number and unit of measurement
☐	☒	A statement on whether measurements were taken from distinct samples or whether the same sample was measured repeatedly
☐	☒	The statistical test(s) used AND whether they are one- or two-sided<br>*Only common tests should be described solely by name; describe more complex techniques in the Methods section.*
☒	☐	A description of all covariates tested
☐	☒	A description of any assumptions or corrections, such as tests of normality and adjustment for multiple comparisons
☐	☒	A full description of the statistical parameters including central tendency (e.g. means) or other basic estimates (e.g. regression coefficient) AND variation (e.g. standard deviation) or associated estimates of uncertainty (e.g. confidence intervals)
☐	☒	For null hypothesis testing, the test statistic (e.g. *F*, *t*, *r*) with confidence intervals, effect sizes, degrees of freedom and *P* value noted<br>*Give P values as exact values whenever suitable.*
☒	☐	For Bayesian analysis, information on the choice of priors and Markov chain Monte Carlo settings
☒	☐	For hierarchical and complex designs, identification of the appropriate level for tests and full reporting of outcomes
☒	☐	Estimates of effect sizes (e.g. Cohen's *d*, Pearson's *r*), indicating how they were calculated

Our web collection on statistics for biologists contains articles on many of the points above.

Software and code

Policy information about availability of computer code

Data collection	Data collection<br>Measurement m6A level:<br>MS/MS was then conducted on the coupled Agilent 6460 Triple Quadrupole (QQQ) mass spectrometer equipped with an Agilent JetStream ESI source which was set to the following parameters: gas temperature, 350 °C; gas flow, 8 l/min; nebulizer pressure, 50 psi; sheath gas temperature, 350 °C; sheath gas flow, 12 l/min; and capillary voltage, 3,000 V<br><br>RNA-seq:<br>The libraries were sequenced on a NextSeq500 with a read length of 85 bp single read and converted to fastq using bcl2fastq (v.2.19) and mapped against Ensembl release 90 of Drosophila melanogaster using STAR (v. 2.5.10)
Data analysis	Data Analysis<br>Proteome label free:<br>Analysis of label free samples was performed using default setting for the LFQ analysis in MaxQuant (1.5.2.8) and the Perseus software version 1.5.6.0 to perform calculation of p-value and Student's T-Test.<br><br>Proteome/Uniquitylome SILAC<br>For peptide identification in SILAC samples, raw data files were analyzed using MaxQuant (development version 1.5.2.8) to calculate the ratios between the different conditions.

RNA-seq:
Counts per gene were derived using featureCounts (v. 2.5.1), Differential expression analysis was performed using Bioconductor/DESeq2 (v1.16.1)) and filtered for an FDR < 5%, default independent filtering was used. Differential splicing analysis was performed using rMATS (v. 3.2.5) and filtered for an FDR < 10%.

For manuscripts utilizing custom algorithms or software that are central to the research but not yet described in published literature, software must be made available to editors and reviewers. We strongly encourage code deposition in a community repository (e.g. GitHub). See the Nature Research guidelines for submitting code & software for further information.

Data

Policy information about availability of data

All manuscripts must include a data availability statement. This statement should provide the following information, where applicable:

- Accession codes, unique identifiers, or web links for publicly available datasets
- A list of figures that have associated raw data
- A description of any restrictions on data availability

All data needed to evaluate the conclusions in the paper are present in the paper and/or Supplementary Materials. Additional data related to this paper may be requested from the authors.
RNA-seq data are available in GEO (GSE158663: https://www.ncbi.nlm.nih.gov/geo/query/acc.cgi?acc=GSE158663).
Proteomics data are available in PRIDE (PXD022294: https://www.ebi.ac.uk/pride/archive/projects/PXD022294).
Source data are provided with this paper.
All data needed to evaluate the conclusions in the paper are present in the paper and/or Supplementary Materials. Additional data related to this paper may be requested from the authors.
RNA-seq data are available in GEO (GSE158663: https://www.ncbi.nlm.nih.gov/geo/query/acc.cgi?acc=GSE158663).
Proteomics data are available in PRIDE (PXD022294: https://www.ebi.ac.uk/pride/archive/projects/PXD022294).
Source data are provided with this paper.

Field-specific reporting

Please select the one below that is the best fit for your research. If you are not sure, read the appropriate sections before making your selection.

☒ Life sciences ☐ Behavioural & social sciences ☐ Ecological, evolutionary & environmental sciences

For a reference copy of the document with all sections, see nature.com/documents/nr-reporting-summary-flat.pdf

Life sciences study design

All studies must disclose on these points even when the disclosure is negative.

Sample size	No sample size calculations were performed. Sample sizes were determined based on our previous studies (PMID: 27919077, 29535189).
Data exclusions	No data were excluded from the analysis
Replication	Experiments were performed by a minimum of two independent replicates as described figure caption or materials and methods sections.
Randomization	Samples were grouped according to genotype (wildtype or various mutants).
Blinding	The investigators were not blinded during data collection as the biological groups (genotypes) were well defined and handled in parallel. Computational analysis was performed by data scientists different from the researchers who collected the data.

Reporting for specific materials, systems and methods

We require information from authors about some types of materials, experimental systems and methods used in many studies. Here, indicate whether each material, system or method listed is relevant to your study. If you are not sure if a list item applies to your research, read the appropriate section before selecting a response.

Materials & experimental systems

n/a	Involved in the study
☐	☒ Antibodies
☐	☒ Eukaryotic cell lines
☒	☐ Palaeontology and archaeology
☐	☒ Animals and other organisms
☒	☐ Human research participants
☒	☐ Clinical data
☒	☐ Dual use research of concern

Methods

n/a	Involved in the study
☒	☐ ChIP-seq
☒	☐ Flow cytometry
☒	☐ MRI-based neuroimaging

Antibodies

Antibodies used

1) mouse anti-Myc 1:2000 (clone 9E10, Enzo (ENZ-ABS462-0200));
2) mouse anti-HA 1:1000 (clone 16B12, COVANCE (MMS-101R));
3) mouse anti-Tubulin 1:2000 (clone TU27, Biolegend (903401));
4) mouse anti-Fl(2)d 1:500 (#9G2, DSHB)
5) guinea pig anti-Mettl3 1:500 (Eurogentec; (Lence et al. 2016, PMID: 27919077))
6) rabbit anti-Mettl14 (Eurogentec; (Lence et al. 2016, PMID: 27919077))
7) rabbit Anti VIRMA antibody 1:1000 (Bethyl A302-124A-M)
8) rabbit Anti HAKAI Antibody 1:1000 (Bethyl A302-969A-M)
9) rabbit RBM15 antibody 1:1000 (Proteintech 10587-1-AP)
10) mouse GAPDH antibody 1:10000 (clone 1E6D9; Proteintech 60004-1-Ig)

Validation

Validated by manufacturers:
1) mouse anti-Myc (#9E10, Enzo); Species reactivity: Human. Specificity: Recognizes epitope sequence EQKLISEEDL, located in aa 410-419 of human c-myc protein. Applications: IF, IHC (FS), IHC (PS), IP, WB
2) mouse anti-HA (#16B12, COVANCE); Antibody Type: Monoclonal. Host Species: Mouse. Immunogen: Monoclonal antibody HA.11 was raised against the twelve amino acid peptide CYPYDVPDYASL. Application: WB - Quality tested; ICC, IP - Verified; FC, Purification - Reported in the literature, not verified in house
3) mouse anti-Tubulin (clone Tu27, Biolegend); Reactivity: Bovine, Rat, Chinese Hamster, Sea Urchin, Chlamydamonas, Drosophila. Antibody Type: Monoclonal. Application: WB, ICC, IHC
4) mouse anti-Fl(2)d (#9G2, DSHB). Antigen Species: Drosophila. Positive Tested Species Reactivity: Drosophila. Recommended Applications: Immunohistochemistry, Immunoprecipitation, Western Blot
5) rabbit Anti VIRMA antibody (Bethyl A302-124A-M). Reactivity: Human. Applications: WB, IP. Antibody Type: Polyclonal. Immunogen: between 1760 and 1810
6) rabbit Anti HAKAI Antibody. Reactivity: Human, Mouse. Applications: WB, IP. Antibody Type: Polyclonal. Immunogen: between 441 and 491
7) rabbit RBM15 antibody (Proteintech 10587-1-AP). Tested Reactivity: Human, Mouse, Rat. Immunogen: RBM15 fusion protein Ag0938. Applications: WB, RIP, IP, IHC, IF, CoIP,ELISA
10) mouse GAPDH antibody (Proteintech 60004-1-Ig). Applications: WB, IP, IHC, IF, FC, CoIP, ChIP,ELISA. Tested Applications Positive WB detected in HeLa cells, soybean whole plant tissue, arabidopsis whole plant tissue, HepG2 cells, ROS1728 cells, pig brain tissue, zebrafish tissue, whole yeast cells, whole Nematode tissue, HEK-293 cells, HSC-T6 cells, PC-12 cells, NIH/3T3 cells, C2C12 cells, SP2/0 cells, rat brain tissue, mouse brain tissue. Positive IP detected in HeLa Cells. Positive IF detected in Ethacrynic acid treated HeLa cells. Positive FC detected in HeLa cells

Validation by gene specific KOs in PMID: 27919077
1) guinea pig anti-Mettl3 1:500 and (Lence et al. 2016)
2) rabbit anti-Mettl14 (Lence et al. 2016)
For anti-Mettl3 sera guinea pig was immunized with a 14 aminoacid-long peptide (163–177 amino acids (AA)); for anti-Mettl14 sera rabbit was immunized with a 14 amino acid-long peptide (240–254 AA).

Eukaryotic cell lines

Policy information about cell lines

Cell line source(s)

S2R+ cells lines were procured from Drosophila Genomics Resource Center, Hela and U2OS cells are commercially available cells lines from CR-UK Cell Services

Authentication

The cell lines were not authencicated because they are commercially available

Mycoplasma contamination

Routinely checked for the presence of Mycoplamsa and the cells lines used in the study were Mycoplamsa free.

Commonly misidentified lines
(See ICLAC register)

No cell lines from the ICLAC register were used.

Animals and other organisms

Policy information about studies involving animals; ARRIVE guidelines recommended for reporting animal research

Laboratory animals	Drosophila melanogaster CantonS flies were used for controls. Mutant lines are described in details in the method section. Animals were not separated by sex
Wild animals	No wild animals were used in this study.
Field-collected samples	No field-collected samples were used in this study.
Ethics oversight	This study did not require an ethical approval.

Note that full information on the approval of the study protocol must also be provided in the manuscript.
